# Gene expression analysis of drought tolerance and cuticular wax biosynthesis in diploid and tetraploid induced wallflowers

**DOI:** 10.1186/s12870-024-05007-6

**Published:** 2024-04-25

**Authors:** Fazilat Fakhrzad, Abolfazl Jowkar

**Affiliations:** Department of Horticultural Science, College of Agriculture, Shiraz University, P.O. Box: 71441-13131, Shiraz, Iran

**Keywords:** Autotetraploid, Cuticular wax compositions, Drought tolerance, *Erysimum cheiri*, Gene expression analysis, Wallflower

## Abstract

Whole-genome doubling leads to cell reprogramming, upregulation of stress genes, and establishment of new pathways of drought stress responses in plants. This study investigated the molecular mechanisms of drought tolerance and cuticular wax characteristics in diploid and tetraploid-induced *Erysimum cheiri*. According to real-time PCR analysis, tetraploid induced wallflowers exhibited increased expression of several genes encoding transcription factors (TFs), including *AREB1* and *AREB3*; the stress response genes *RD29A* and *ERD1* under drought stress conditions. Furthermore, two cuticular wax biosynthetic pathway genes, *CER1* and *SHN1*, were upregulated in tetraploid plants under drought conditions. Leaf morphological studies revealed that tetraploid leaves were covered with unique cuticular wax crystalloids, which produced a white fluffy appearance, while the diploid leaves were green and smooth. The greater content of epicuticular wax in tetraploid leaves than in diploid leaves can explain the decrease in cuticle permeability as well as the decrease in water loss and improvement in drought tolerance in wallflowers. GC‒MS analysis revealed that the wax components included alkanes, alcohols, aldehydes, and fatty acids. The most abundant wax compound in this plant was alkanes (50%), the most predominant of which was C29. The relative abundance of these compounds increased significantly in tetraploid plants under drought stress conditions. These findings revealed that tetraploid-induced wallflowers presented upregulation of multiple drought-related and wax biosynthesis genes; therefore, polyploidization has proved useful for improving plant drought tolerance.

**Supplementary Information:**

The online version contains supplementary material available at 10.1186/s12870-024-05007-6.

## Background

Plants encounter an extensive range of biotic and abiotic stresses that threaten plant growth and productivity. Drought is the major environmental stress affecting plant growth and metabolism. To adapt to drought stress conditions, plants have evolved a variety of mechanisms, including avoidance, escape, and tolerance, through triggering molecular and cellular pathways to achieve physiological adaptations, such as deep root systems; stomatal movement mediated by ABA-mediated movement of stomata; leaf morphology changes; cuticular wax thickening; and cutinization of the leaf surface [1]. The response to drought stress relies on the expression of a network of stress-responsive genes and transcription factors (TFs) that regulate the expression of stress genes [2]. It is well known that genetic manipulations such as ploidy induction affect the expression of genes involved in stress signaling pathways [1]. Polyploidization enhances plant adaptability to adverse environmental stresses [3]. Recently, some studies have indicated that polyploidy in *Miscanthus lutarioriparius* [4], *Morus alba* [5] and *Erysimum cheiri* [6] enhances stress tolerance compared with that of their diploid counterparts. Improved tolerance and adaptation to stress conditions in polyploids may be caused by gene dosage effects or epigenetic changes such as DNA methylation, histone modifications, or changes in splicing patterns after doubling of the whole genome [7]. Wei et al. [8] compared the transcriptome profiles of tetraploid-induced and diploid plants of *Poncirus trifoliata*. These authors revealed that genes involved in the antioxidant system and sugar metabolism were upregulated in tetraploid plants. In another study, chromosome-doubled *Citrus limonia* plants exhibited increased expression of ABA and other signaling genes, with greater adaption to drought stress; and further concluded that polyploidization may cause changes in the expression of stress tolerance genes [9]. Moreover, Zhang et al. [10] reported that DNA methylation was responsible for the expression of stress-related genes in polyploidized rice. Xu et al. [4] reported that tetraploid *Miscanthus lutarioriparius* can tolerate drought conditions by upregulating a series of drought-responsive genes and/or by activating other drought-related pathways, while diploid plants mitigate drought stress by regulating the expression of only some drought-related genes.

The limitation of cuticular transpiration is one of the most important evolutionary adaptation mechanisms for the survival and growth of plants under water deficit conditions. Cuticular waxes, as the most important component of the cuticle, cover all aerial parts of plants and protect plants from nonstomatal water loss, UV radiation damage, and bacterial and fungal invasion. Therefore, this layer plays a very important role in increasing plant tolerance to biotic and abiotic stresses. Cuticular wax consists of a hydrophobic aliphatic mixture of very long-chain fatty acids (VLCFAs) that are derived from primary and secondary alcohols, aldehydes, ketones, esters, and lower amounts of triterpenoids, sterols, polyketides, and flavonoids [11]. The cuticular wax load and composition can vary depending on the stage of development, plant organ, and abiotic and biotic factors [1, 11].

In recent years, epidermal wax synthesis-related genes and transcription factors (TFs) involved in wax biosynthesis pathways have been widely cloned and studied. Overexpression of these genes alters cuticular wax accumulation and/or composition, often enhancing stress tolerance [12]. Whole-genome doubling changes the content and composition of cuticular waxes by affecting the gene dosage and changing several hormone and metabolic pathways related to stress. However, the mechanisms of stress tolerance caused by the genetic dosage effect are unknown [13]. Recent studies have reported triploid mulberry [5] and tetraploid sour jujube plants [14], which have higher cuticular wax contents and some changes in wax composition, compared to diploid plants.

Wallflower (*Erysimum cheiri* (L.) Crantz) (2*n* = 2*x* = 12) is popular as a flowering pot or bedding plant with ornamental and medicinal values [15]. This plant is suitable for using in rock gardens and xeriscapes due to its tolerance to water deficit conditions. Although limited studies have been done so far, this plant has the capability to be used as a cut flower in ornamental plants industry by genetic manipulation and creating traits such as double flowering and more fragrance in addition to further drought tolerance and longer vase life. Also, the biosynthesis mechanisms of secondary and medicinal metabolites of this plant with anti-inflammatory, anti-tumor and cardioactive properties need comprehensive investigations. In a previous study, the drought tolerance of autotetraploid-induced wallflowers was reportedly greater than that of diploids, and autotetraploid-induced wallflowers presented significant increases in the levels of antioxidant enzymes, phytochemicals, secondary metabolites, and stress-related phytohormones [6]. Gene expression changes are crucial for regulating physiological and biological processes, including the abiotic stress response. This study was conducted to better understand the molecular mechanisms responsible for drought tolerance by comparing the gene expression patterns of drought tolerance and biosynthesis of cuticular wax and investigating the morphological, physiological, and metabolic properties of cuticular wax from tetraploid and diploid wallflower leaves.

## Materials and methods

### Plant material, treatments and experimental design

The diploid and tetraploid induced wallflowers [16] (6 months old) were used as plant samples. All the plants were grown in media supplemented with peat: perlite: loamy soil (1:1:1) and kept in a greenhouse at a temperature of 24/20 ± 2 °C and a natural photoperiod. The plants were subjected to well-watered (100% field capacity) and water deficit (50% field capacity) conditions for one month. Young upper leaves from each treatment were taken as plant samples, and three biological replicates were snap-frozen in liquid nitrogen for use in the gene expression assay. For physiological and biochemical assays and microscopic observations, five biological replicates of fully expanded leaves were collected from diploid and tetraploid plants.

### RNA extraction, cDNA synthesis and quantitative real-time PCR

RNA was extracted from the leaves of diploid and tetraploid-induced plants under control (100% FC) and water deficit (50% FC) conditions using a kit (Denazist, Iran), according to the protocol. The purity and quantity of the RNAs were evaluated using a NanoDrop (ND) 1000 spectrophotometer (Wilmington, USA). Agarose gel electrophoresis (1%) was used to determine the integrity of the isolated RNA. Before cDNA synthesis, DNase (Yektatajhiz, Tehran, Iran) was applied to remove genomic DNA, and cDNA was synthesized based on the instructions of the cDNA kit (Yektatajhiz, Tehran, Iran). In this study, conserved regions in each gene were identified through BLAST (NCBI) in *Arabidopsis* as the model plant in the Brassicaceae family, and the primer sequences were designed using Allele ID 7.5 software (Table S1). The ABI Step One ver. 2.3 (Applied Biosystems, USA) was used for real-time PCR assays with a 20 µL reaction mixture containing 5 µL of cDNA template, 10 µL of SYBR Green (RealQ Plus Master Mix Green, AMPLIQON, Denmark), 1 µL of each primer (forward or reverse primer) and 3 µL of water. The amplification procedure included an initial denaturation step of 95 °C for 10 min, followed by 40 cycles of 95 °C for 15 s, a dedicated annealing temperature for each gene (Table S1) for 30 s, and a final extension at 72 °C for 30 s. The relative gene expression levels were calculated with the 2^−ΔΔCt^ formula [17] using *ACTIN2* as an internal reference gene. Three replicates of both biological and technical samples were analyzed for gene expression.

$${\rm{Fold}}\,{\rm{change}}\,{\rm{ = }}\,{{\rm{2}}^{{\rm{ - \Delta \Delta Ct}}}}$$

Where Ct is the threshold cycle value; ΔCt is the difference between Ct of the target gene and Ct of the reference gene; and ΔΔCt is the difference between ΔCt of the treatment samples and ΔCt of the control samples.

### Scanning electron microscopy (SEM)

To observe cuticular wax crystalloids, five leaves from each diploid and tetraploid plant under control and drought treatment conditions were collected and air-dried at room temperature. Microscopic observation was performed using a TESCAN-Vega 3 scanning electron microscope (Brno, Czech Republic) [18].

### Cuticular wax content

To measure the cuticular wax contents, a total of 5 leaves per treatment were immersed in 5 ml of chloroform for 1 min. The chloroform was then evaporated at 70 °C overnight. The wax content (µg/cm^2^) was calculated as the microgram of wax left in the test tube per two sides of the leaf area in cm^2^ [14].

### Water loss rate

Five leaves were sampled from well-watered and stressed diploid and tetraploid wallflowers. The leaves were immersed in distilled water for 3 h in the dark and then weighed every 30 min for 300 min (at room temperature and approximately 70% humidity). The leaves were then oven-dried for 24 h at 70 °C. The water loss rate was measured as the percentage of the water lost in the water-saturated initial leaf weight [19].

### Cuticular wax composition

Lipid extracts of diploid and tetraploid *Erysimum* leaves grown under water deficit and control conditions (10 pooled leaves for each sample) were prepared before gas chromatography‒mass spectrometry (GC‒MS) analysis according to the methods of Loneman et al. [20]. For each sample, 0.5 mg of leaf was shaken with 2 ml of chloroform for 1 min. To evaporate the chloroform, the mixture was incubated at 40 °C overnight. After adding 2 ml of methanolic acid (1 N), the mixture was incubated at 75 °C for 1 h. Two milliliters of aqueous NaCl (0.9% (w/v)) were added, and the mixture was vortexed in 1 ml of hexane at 2500 rpm for 1 min. After centrifugation at 1,000 rpm for 5 min, the organic layer was recovered. To enhance the amount of recovered lipids, the supernatant layer was extracted two times with hexanes, and the recovered extracts were pooled. The samples were dried under a stream of N_2_ gas. The cuticular wax composition was analyzed via GC–MS with an HP-5MS column (30 m in length; 0.25 mm inner diameter) using an Agilent Technologies series 7890B gas chromatograph equipped with a model 5977 A mass detector. Helium was used as the carrier gas at a flow rate of 1.0 ml/min. The oven temperature program was set to increase from 60 to 280 °C as follows: 60 °C for 1 min, increase to 140 °C at a rate of 20 °C/min, reach 280 °C at a rate of 5 °C/min and maintain at this temperature for 15 min. The wax composition was identified by comparing the retention times with those described in the Wily 7n and NIST05a libraries. To quantify the wax components, hexadecane was used as the internal standard.

### Experimental design and data analysis

The data were analyzed using SAS software version 9.4 (SAS Institute, USA), and the LSD test at *p* ≤ 0.05 was used for mean comparisons.

## Results

### Relative gene expression

**Fig. 1:**
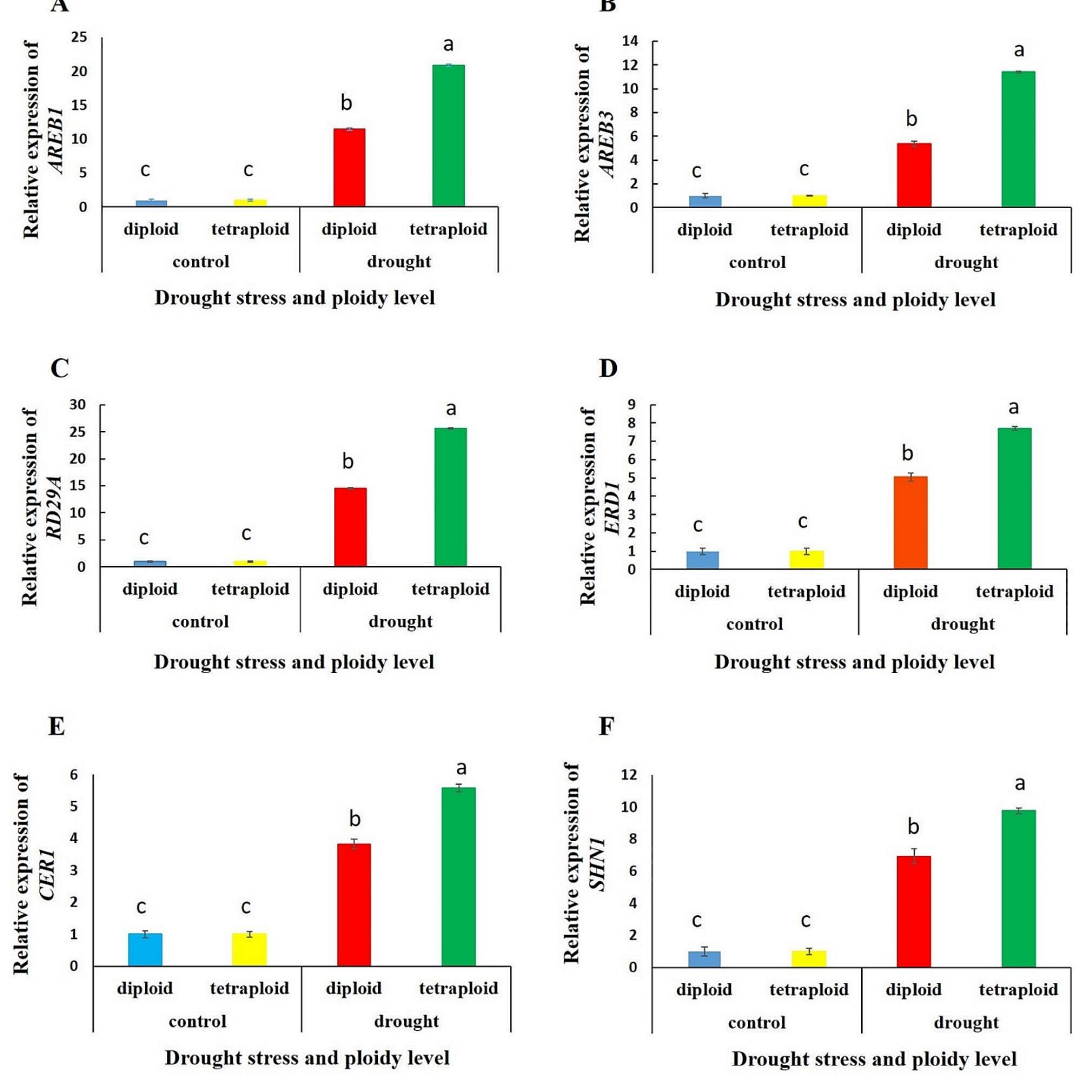
Relative gene expression levels of diploid and tetraploid wallflowers under control (100% FC) and water deficit (50% FC) conditions; (**A**) *AREB1*, (**B**) *AREB3*, (**C**) *RD29A*, (**D**) *ERD1*, (**E**) *CER1* and (**F**) *SHN1*. The data are presented as the means of three biological replicates with three technical replicates each. The error bars are the means ± SDs. Different letters indicate significant differences at *p* ≤ 0.05 according to the LSD test

In this study, the expression of several genes and transcription factors related to drought tolerance and cuticular wax biosynthesis, *AREB1* and *AREB3* (ABA-responsive cis-element binding protein), *RD29A* (responsive to dehydration), *ERD1* (early responsive to dehydration), *CER1* (*eceriferum1*), and *WIN1*/*SHN1* (*WAX INDUCER 1*/*SHINE1*), was evaluated. The results of *AREB1* gene expression showed a highly great increase in the expression of this gene in both diploid (11 folds) and tetraploid (20.9 folds) plants under water stress. Tetraploid-induced plants expressed this gene approximately 82% more highly than diploids under water deficit conditions. *AREB3* gene expression in 2*x* and 4*x* plants exposed to water stress was 5.4- and 11.4-fold greater, respectively, than that in plants fully irrigated with water. *AREB3* expression was upregulated 113% more in these plants than in diploid plants (Fig. 1. B). The patterns of *AREB1* and *AREB3* gene expression were similar in the drought-stressed wallflowers. The largest increase in the expression level of these genes occurred in the tetraploid-induced plants under water deficit conditions (Fig. 1. A). When polyploidized plants were exposed to water stress, the expression of the *RD29A* gene increased 25.6-fold, while 2*x* wallflowers upregulated this gene 14.5-fold. The *RD29A* gene was expressed more in tetraploid plants (76.5%) than in diploid plants (Fig. 1. C). The expression of the *ERD1* gene was also increased in both tetraploid (7.7-fold) and diploid plants (5-fold) under drought-stress conditions. 4*x* wallflowers expressed this gene 53% more than diploids under water stress (Fig. 1. D). Diploid and tetraploid plants prone to water scarcity exhibited significantly greater *CER1* gene expression than did the control plants (by 7- and 10-fold, respectively). Compared with diploids, 4*x* plants presented a 40.69% increase in *CER1* gene expression under water stress conditions. (Fig. 1. E). Under drought, the expression of *SHN1* in tetraploid plants was upregulated by 57% compared with that in diploids. The normal and tetraploid induced wallflowers expressed this gene more under water deficit conditions (4 and 6 times, respectively) than under well-watered conditions (Fig. 1. F).

### Epidermal appearance and SEM analysis of leaf epicuticular wax

**Fig. 2:**
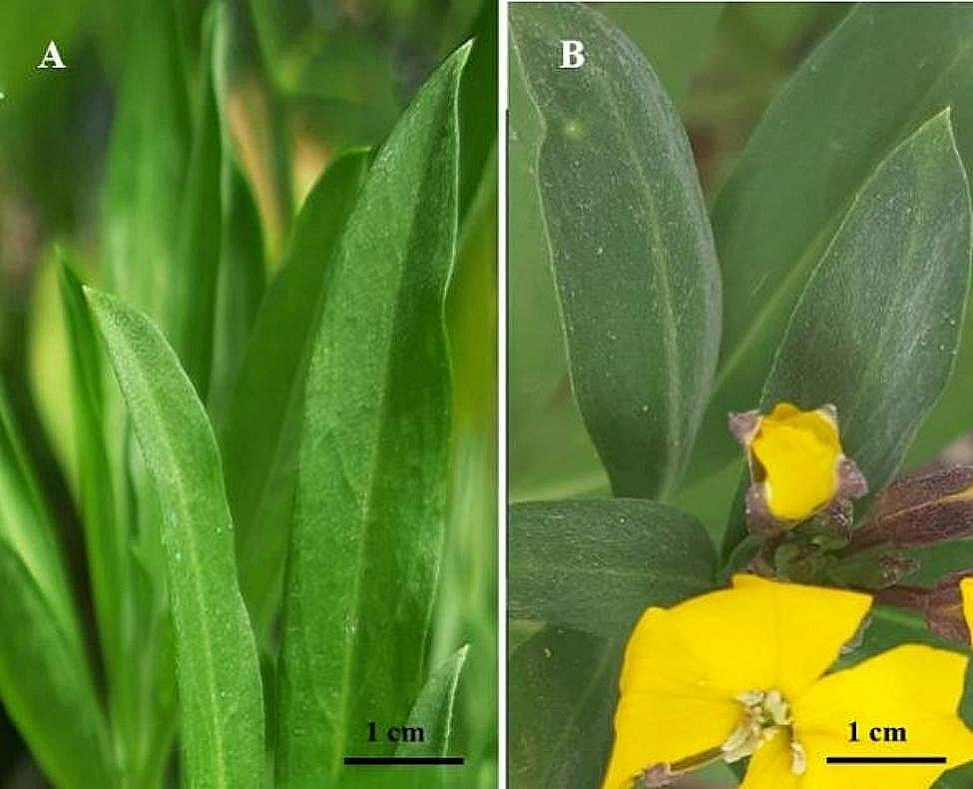
The effect of water deficit conditions on the epidermal appearance of *E. cheiri* leaves. (**A**) Smooth and light green diploid leaves. (**B**) Rough and dark green tetraploid induced leaves covered with powdery wax

The 2*x* and 4*x* leaves of the wallflowers were phenotypically evaluated under drought conditions. The tetraploid leaves were rough and dark green and covered with white powdery wax, while the diploid plants had smooth and light green leaves without powdery wax (Fig. 2. A and B).

**Fig. 3:**
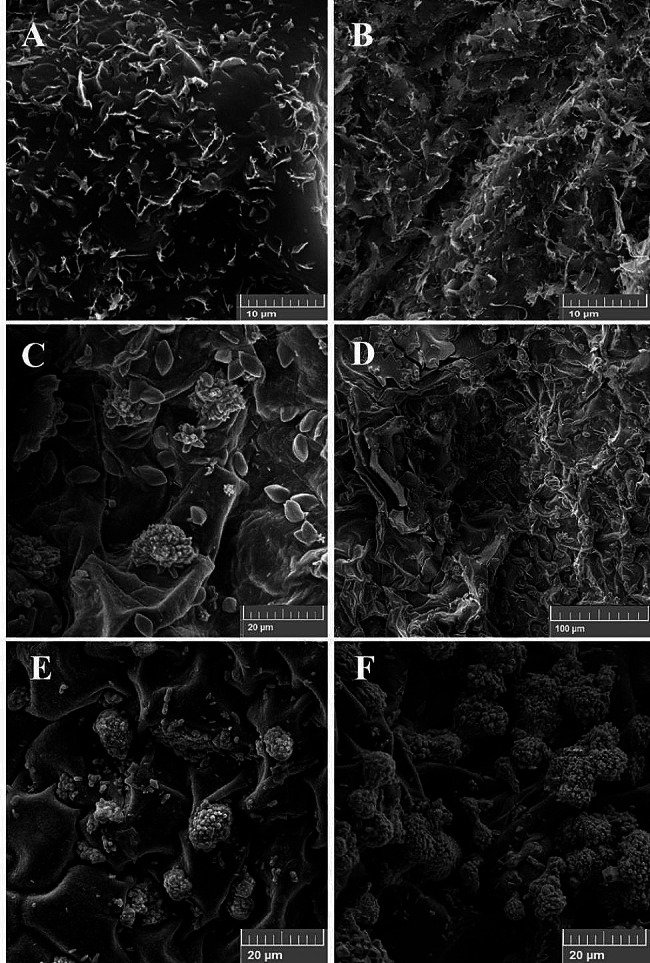
Scanning electron microscopy images of the density of wax platelets on the leaf epidermis of (**A**) diploid and (**B**) tetraploid induced *Erysimum* under drought conditions; (**C**) and (**D**) Formation of single wax rodlets, polygonal rodlet structures, and tubular clusters on the leaf surface of tetraploid wallflowers under drought stress conditions; Density of tubular clusters of wax on the leaf epidermis of tetraploid induced *E. cheiri* under (**E**) full irrigation and (**F**) water stress conditions

Under full irrigation conditions, less phenotypic difference was observed in terms of wax content between diploid and tetraploid leaves. The epicuticular wax of diploid and tetraploid *Erysimum* leaves exhibited great micromorphological diversity under well-watered and water stress conditions. The crystalloid wax was different in terms of density, shape, and size. The predominant form of leaf cuticular wax was an amorphic film layer on top of the epidermis, in which platelets with irregular arrangement were visible (Fig. 3. A and B).

Compared with that in diploid leaves, the density of wax platelets was significantly greater in 4*x* plants (Fig. 3. A and B). On the basis of microscopic observations, different specific structures, including single wax rodlets, polygonal rodlet structures, and tubular clusters, were observed in addition to platelets in tetraploid plants but not on the leaf surface of diploids. As shown in Fig. 3 (C and D), the tubular clusters of tetraploid wallflowers were arranged into clusters resembling rock crystals. There was a denser accumulation of tubular clusters and polygonal rodlet structures under water deficit conditions than under well-watered conditions. These tetraploid-specific structures were less common under fully irrigated conditions (Fig. 3. E and F).

### Cuticular wax content

**Fig. 4:**
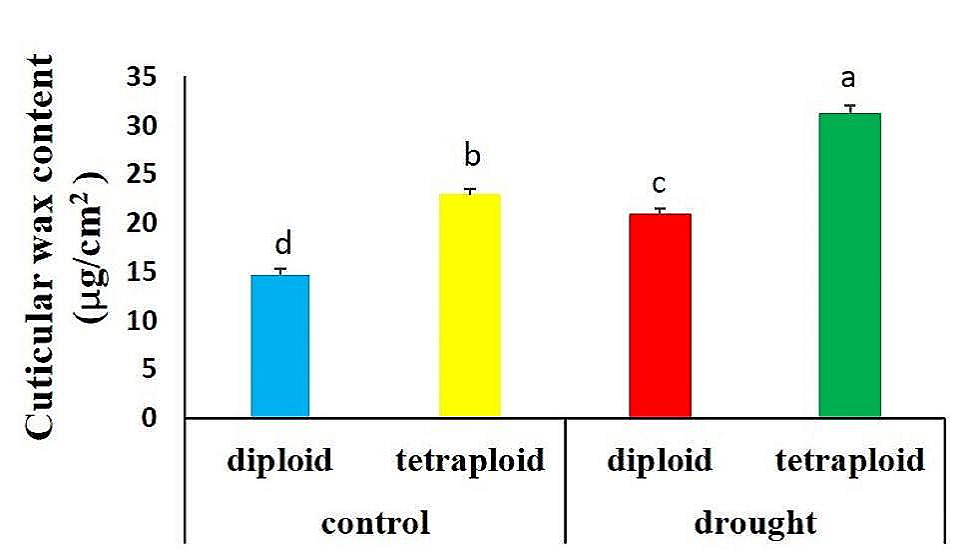
The cuticular wax content of diploid and tetraploid wallflower leaves grown under control and drought conditions. The values are presented as the means ± SEs. Different letters indicate significant differences according to LSD at *p* ≤ 0.05

The maximum cuticular wax content was observed in tetraploid plants under water stress (31.20 µg/cm^2^), while the minimum amount of wax was seen in diploid leaves under full irrigation (14.70 µg/cm^2^). Tetraploid leaves contained more wax under both water stress (50% FC) and well-watered (100% FC) conditions than diploids under similar conditions (49.28% and 55.78%, respectively). After drought stress, the total epicuticular wax content of diploid and tetraploid genotypes increased by 42.17% and 36.24%, respectively (Fig. 4).

### Water loss rate

**Fig. 5:**
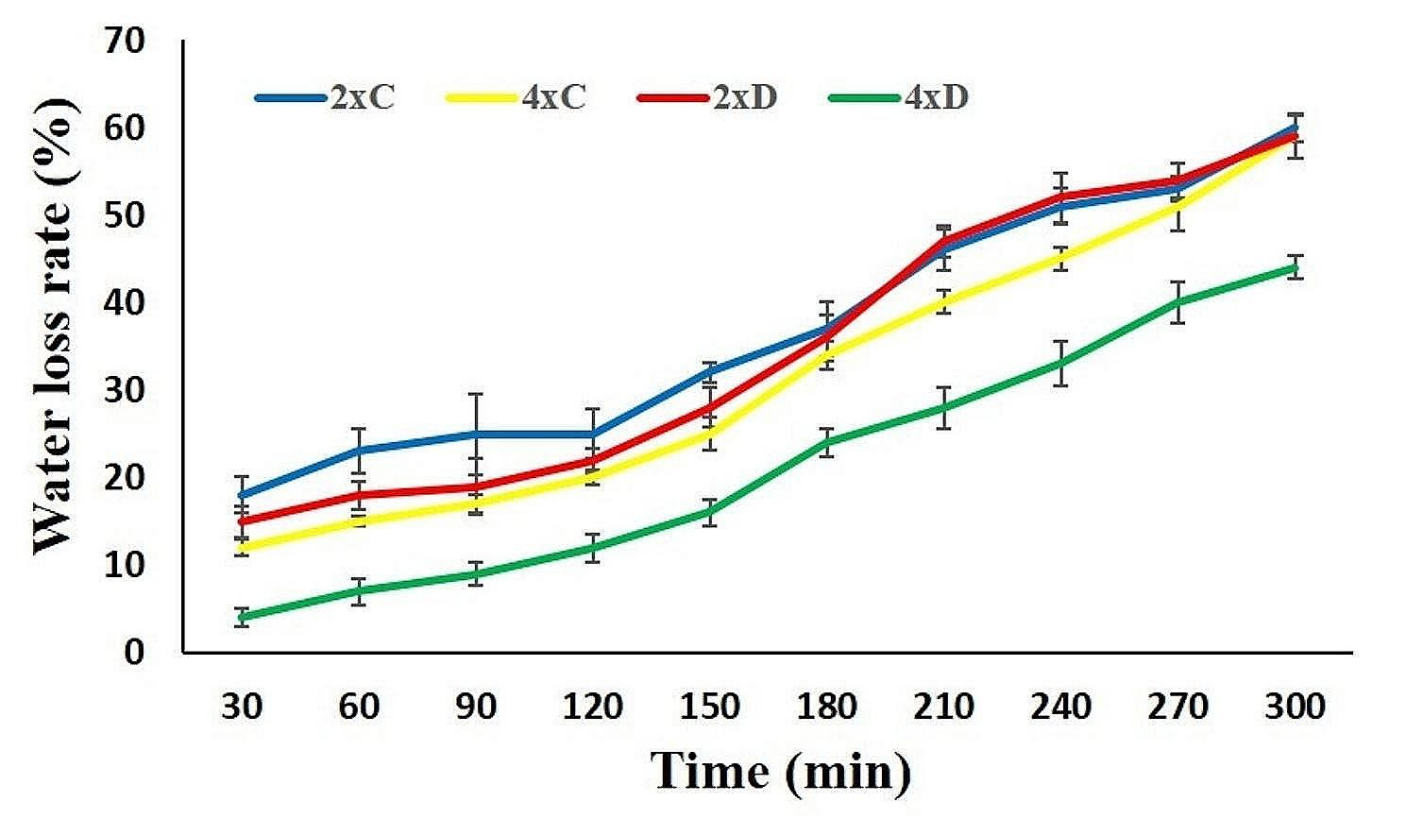
Water loss rate of diploid and tetraploid wallflower leaves under water deficit (50% FC) and well-watered (100% FC) conditions. **2*x*C**: diploid plants under well-watered control condition; **2*x*D**: diploid plants under drought stress; **4*x*C**: tetraploid plants under well-watered control condition; **4*x*D**: tetraploid plants under drought stress. The values are presented as the means ± SEs

The average water loss rate of the tetraploid leaves was lower (21% and 32%) than that of the diploid plants (35% and 37%) under both drought and control conditions, respectively. The water loss in both drought-treated and fully irrigated diploids increased at a constant rate until 120 min and then increased substantially (Fig. 5). Generally, tetraploid wallflowers indicated lowest water loss rate during experiment.

### Cuticular wax components

**Fig. 6:**
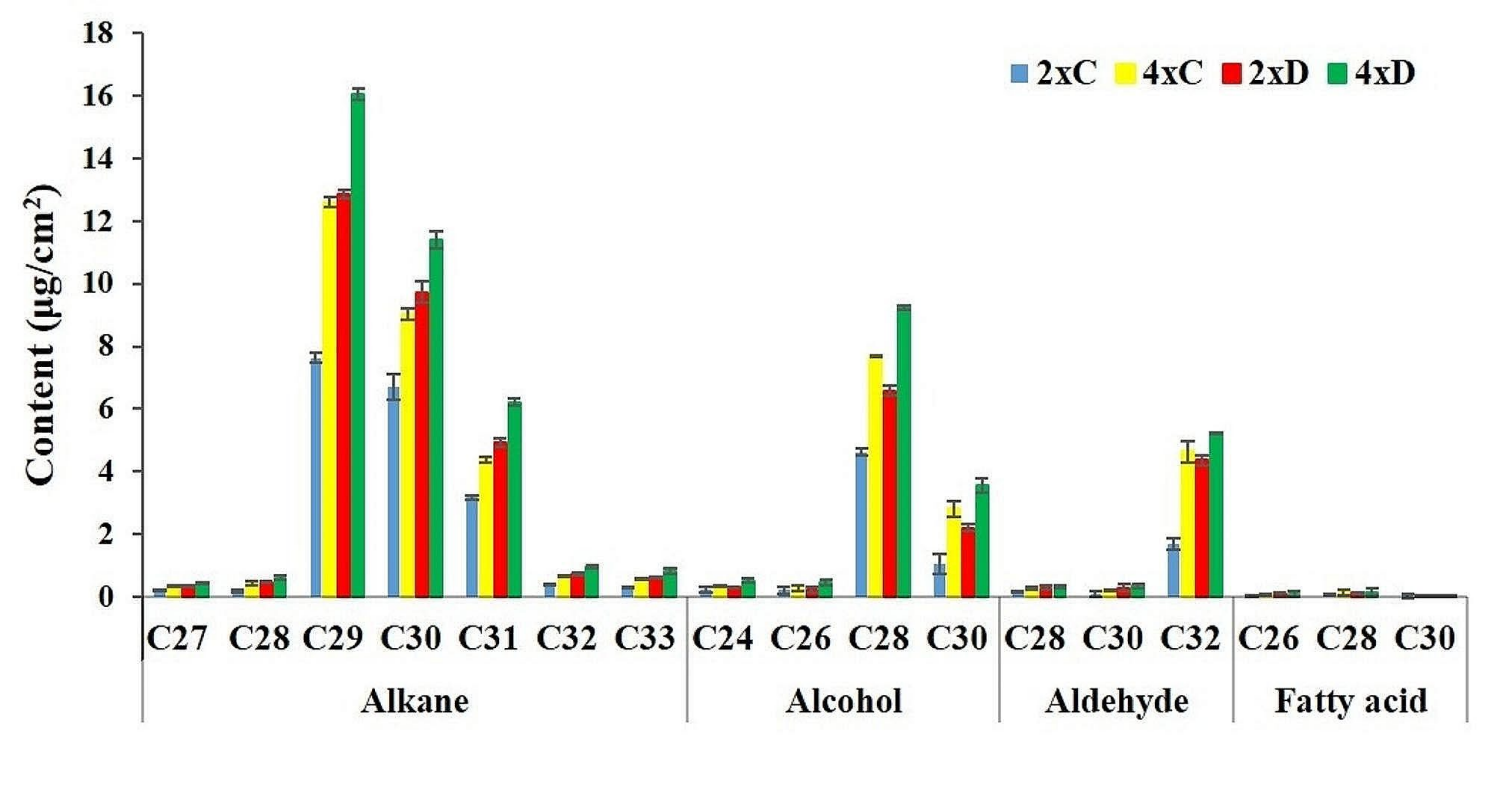
The composition of cuticular wax of diploid and tetraploid *E. cheiri* under drought stress (50% FC) and well-watered control (100% FC) conditions. **2*x*C**: diploid plants under well-watered control conditions; **2*x*D**: diploid plants under drought stress; **4*x*C**: tetraploid plants under well-watered control conditions; **4*x*D**: tetraploid plants under drought stress. Each value indicates the mean of three replications (± SD)

GC–MS analysis revealed four different chemicals in the wax collected from all the treatment groups (Fig. S1). These components include alkanes (50%), alcohols (31%), aldehydes (18%), and fatty acids (1%). Alkane was the most common component of the cuticular wax of leaves; among these components, C29-alkane (43%) was the most common, whereas C28 and C32 carbons were the most prevalent alcohols and aldehydes, respectively. Alkanes appeared to be particularly important in the formation of wax in leaves. The identified alkanes included seven compounds with chain lengths ranging from C27 to C33 (Fig. 6). The two alkanes C29 and C30 together included approximately 76% of the total alkanes in the leaf wax of all the treatments. The alcohol included compounds with carbon atoms in the range of C24 to C30 (Fig. 6). The aldehydic compounds included C28, C30, and C32, and the fatty acids included C26, C28, and C30 (Fig. 6). The leaf wax of tetraploid wallflowers had higher contents of alkanes, alcohols, aldehydes, and fatty acids than did that of diploid plants under water stress conditions (Fig. 6). Furthermore, there were significant differences in the amounts of alkanes (C29 and C30) produced by tetraploid and diploid plants under stress and control conditions. Compared with those under full irrigation, the tetraploid and diploid wallflowers produced 43.78% and 23.28% greater amounts of C29 alkanes and 62.39 and 87.74% more C30 alkanes under stress conditions, respectively. Under water stress conditions, tetraploid plants produced 11.53% more alkanes than diploid plants. Diploid plants under drought stress had 5.65% and 5% more alkane and fatty acids, respectively, than tetraploid plants under control conditions. Conversely, compared with drought-stressed diploids, tetraploid plants under control conditions consisted of a greater content of alcohol (19.23%) and aldehyde (3.63%).

### Heatmap analysis

**Fig. 7:**
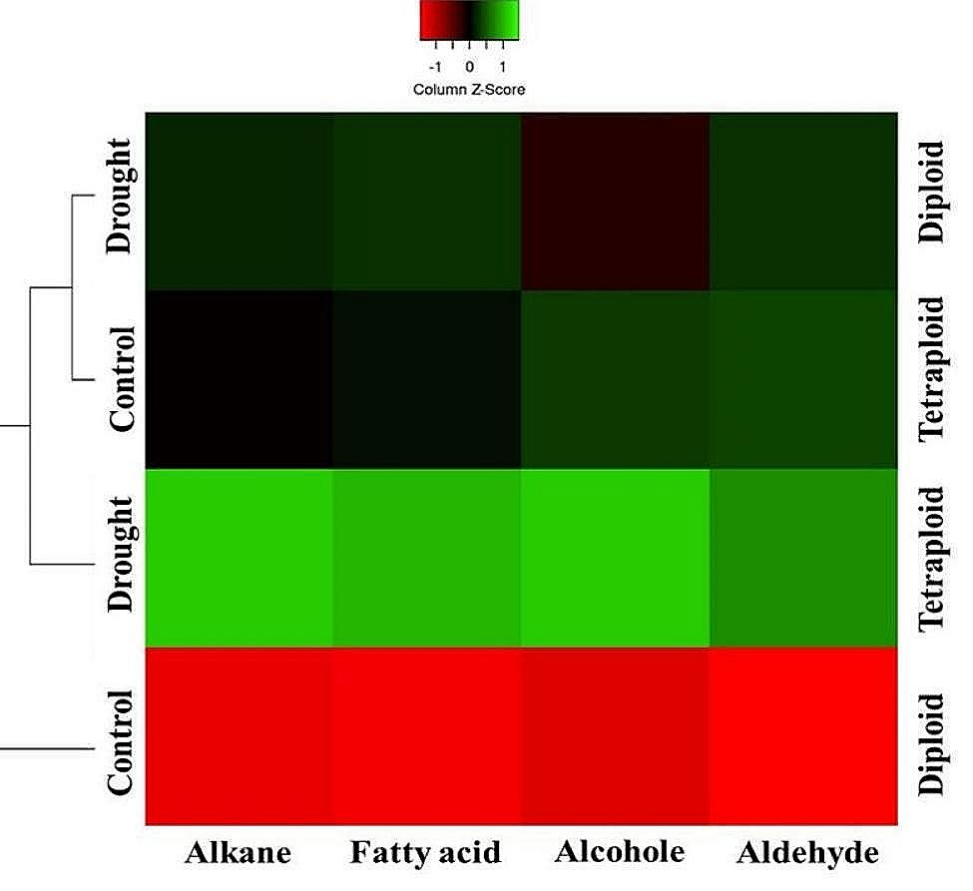
Heatmap of cuticular wax compositions of diploid and tetraploid wallflowers under drought and full irrigation conditions. Green and red indicate higher and lower contents, respectively

The heatmap analysis showed that the biosynthesis of alkanes and fatty acids was more affected by stress than ploidy level. The amounts of alkanes and fatty acids in diploid plants under stress was greater than that in tetraploid plants under control conditions. However, alcohol and aldehyde production were more strongly correlated with increased ploidy levels (Fig. 7).

## Discussion

The increase in genetic diversity caused by whole-genome duplication and gene redundancy caused by differential expression of genes and the creation of a complex regulatory network allows polyploid plants to adapt to adverse environmental conditions. Plant acclimatization against abiotic stresses involves a set of pathways for signal perception and transduction, gene expression, and the synthesis of proteins and stress-related metabolites [21]. Drought-responsive genes can be classified into two main groups: functional genes that directly control stress tolerance, such as genes that encode osmolyte biosynthesis enzymes and detoxification enzymes; and regulatory genes, such as those encoding transcription factors that play a role in signal transduction and the regulation of gene expression [2]. Transcription factors (TFs) are the most important components of transcriptional regulatory and signal transduction systems. These transcription factors are associated with the cis-acting elements of the promoter regions of stress-inducible genes and activate or repress gene expression. These transcription factors usually regulate various stress-responsive genes cooperatively by establishing transcriptional gene networks [22]. In this study, the specific genes were chosen due to their importance for drought tolerance and cuticular wax biosynthesis. These genes play critical roles in both ABA-dependent and ABA-independent signaling pathways, thereby enabling plants to flourish and endure even in water-scarce environments. By scrutinizing the expression patterns and functional attributes of these genes within plants possessing enlarged genomes, it is possible to identify molecular pathways that enhance plant adaptability in response to drought-induced stress.

*AREB/ABF* (ABA-responsive cis-element binding protein/ABA-responsive cis-element binding factor) belongs to the bZIP (basic leucine zipper) transcription factor family, which recognizes and binds to the ABA-responsive cis-element (ABRE; PyACGTGG/TC) in the promoter of ABA- and/or stress-regulated genes [22]. *AREB1*, one of the most important members of this family, is implicated in osmoprotection and antioxidant activity by regulating a set of genes downstream of the ABA signaling pathway and in response to stress. The expression of *AREB1* was reportedly upregulated in *Arabidopsis* [23, 24] and rice [25] under osmotic stress. *AREB3* is another member of this family whose expression is upregulated under stress conditions. Wang et al. [26] showed that *TaAREB3* isolated from wheat can activate downstream target genes (*RD29A*, *RD29B*, (cold regulated) *COR15A*, and *COR47*) by binding to their promoter regions in transgenic *Arabidopsis*. In the present study, the expression of the *AREB1* and *AREB3* genes significantly increased in response to water deficit conditions compared to the control in both diploid and tetraploid plants. Our results confirmed the findings of Wang et al. [26], who reported that the *AREB* proteins and subsequent induction of downstream gene expression were activated via an ABA-dependent pathway under stress conditions. According to Rao et al. [3], polyploidization enhances the function of TFs by changing their regulatory mechanisms. The authors reported that increased ABA content in genome-doubled plants directly led to the induction of SNF1-related protein kinase (*SnRK2*) phosphorylation in *AREB/ABFs*, the upregulation of ABFs and the activation of downstream stress genes, which is an important regulatory mechanism of ABA-activated ABFs [3]. In the present study, both *AREB1* and *AREB3* were significantly more highly expressed in autotetraploids than in diploids under water deficit conditions, which was in line with the findings of Rao et al. [3].

Generally, the target genes mostly encode key enzymes for osmolyte biosynthesis, late embryogenesis abundant proteins (LEAs), and antioxidant enzymes that improve plant stress tolerance [22]. The LEA gene family includes numerous genes, such as *RD, COR, ERD*, and *RAB* (responsive to ABA), which can protect protein integrity and cell structure from drought stress damage [26, 27]. *RD29* includes two closely related genes, *RD29A* and *RD29B*, in the *Arabidopsis* genome that encode very similar proteins [28]. These genes are strongly induced by drought, salt and temperature stresses and appear to be limited to the Brassicaceae family. The *RD29* gene is stress inducible and not expressed under normal conditions [28]. ABA-responsive expression of the *RD29A* gene was shown to require both *DREB* (dehydration responsive element-binding) and *AREB* (interactive transcriptional activators) [29]. Yamaguchi-Shinozaki and Shinozaki [2] confirmed the occurrence of cross-talk between *DRE* (dehydration responsive element) and ABRE in the *RD29A* promoter. In a study by Roca Paixão et al. [23], using the CRISPRa dCas9HAT system, the increased expression of *AREB1* and *RD29A* genes improved drought stress tolerance in transgenic *Arabidopsis* plants. Based on our results, *RD29A* was strongly induced under drought conditions, while both diploid and tetraploid wallflower genotypes exhibited negligible *RD29A* gene expression under normal conditions. Our results are in line with the abovementioned findings that *AREB* TFs can synergistically play an effective regulatory role in increasing *RD29A* gene expression. Furthermore, *RD29A* was highly expressed in tetraploid wallflowers under water deficit conditions, which is an indicator of greater drought tolerance in tetraploids than in diploids.

*ERD1* is an early-response gene in dehydration stress, and its expression is induced by dehydration stress but not by ABA [30]. The *ERD1* promoter includes the recognition sequence *NACRS* and zinc finger homeodomain (*ZFHDR*) [31]. Tran et al. [29] reported that the coexpression of the *ZFHD1* (zinc finger homeodomain 1) and *NAC* (NAM, ATAF, and CUC) genes in the ABA-independent pathway upregulated the expression of *ERD1* and improved drought tolerance. Xu et al. [24] reported that AREBs/ABFs in the ABA-dependent pathway and *NAC*s in the ABA-independent pathway synergistically interact to activate drought-responsive genes such as *RD29A* and *ERD1*. Yu et al. [32] reported that the expression of *ERD1* was promoted by the synergistic regulation of *DREB2A* and *ABF3* in an ABA-dependent signaling cascade controlled by *PwNAC11* in Arabidopsis. Moreover, Li et al. [30] reported that *ERD1* is expressed downstream of myelocytomatosis 2 (*MYC2*) in the jasmonic acid (JA)-mediated signaling pathway. JAZ (jasmonate Zim Domain) proteins play a major role in the JA signaling pathway. Under normal conditions, when JA is present at a very low concentration, JAZ proteins attach to transcription factors, such as MYC2, to minimize their activity. However, under stress conditions, when JA is abundant, the JAZ protein is degraded, and stress-responsive transcription factors are activated, which further regulate the target genes (*ERD1*) involved in the stress response [21]. De Ollas et al. [33] reported that JA can regulate ABA levels in plants under drought-stress conditions. For rapid and strong responses to abiotic stresses, several synergistic interactions between different pathways and TFs are carried out [24]. It seems that polyploidy enhances stress tolerance by creating a complex regulatory network with differentially expressed genes and can enable multi-strategy responses to stress in tetraploids [7]. Under water deficit conditions, the expression level of *ERD1* in tetraploid wallflowers was 7.7-fold greater than that in the control, while diploid plants exhibited 5-fold greater expression of this gene during drought conditions. Our results indicated that, in addition to *RD29A* and *ERD1*, *AREB1* and *AREB3* (two members of the AREBs) were more highly expressed in tetraploid wallflowers than in diploid plants, which may be the reason for the difference in stress tolerance. Overall, the extensive transcriptional reprogramming of a vast number of genes involved in stress responses might activate some molecular and physiological mechanisms pertinent to drought tolerance in tetraploid plants [3, 8].

Increasing the amount of epidermal wax under water deficit conditions is one of the solutions used by plants to prevent nonstomatal water loss. Previous studies have reported that the accumulation of cuticular wax is associated with drought tolerance in many plants, such as *Arabidopsis* [19], sorghum [11], wheat [34] and tobacco [35]. The synthesis and transport of drought-induced ABA cause stomatal closure and induce the expression of many abiotic stress-related genes, including wax biosynthetic genes [1]. During growth, cuticular wax is deposited on plant surfaces, and its amount is regulated by wax genes in response to drought stress. The eceriferum gene family (*CERs*) encodes many enzymes involved in wax biosynthesis pathways. The *CER1* gene encodes aldehyde decarbonylase and plays an important role in wax biosynthesis by catalyzing the conversion of aldehydes to alkanes [36]. This gene is induced by ABA and osmotic stress, whose expression leads to rapid regulation of alkane biosynthesis, cuticle permeability, and drought tolerance [19].

Furthermore, a complex of TFs has been implicated in cuticular wax biosynthesis and accumulation under drought stress [37]. *WIN1*/*SHN1*, which are APETALA2/ethylene-responsive factor (*AP2*/*EREBP*) family members, are the first reported TFs that regulate cuticular wax and cutin biosynthesis by regulating the expression of waxy genes, including the *CER1*, *CER2* and *KCS1* (3-ketoacyl-CoA synthase 1) genes [38]. Interestingly, this TF plays dual roles in both stomatal conductance by altering stomatal features and closing stomata and in nonstomatal conductance through cuticle formation and water loss control [39]. Park et al. [40] reported that the overexpression of *AtSHN* alters epidermal characteristics and increases the wax content of the *Arabidopsis* epidermis. Moreover, *SHN1* overexpression led to a greater wax content, enhanced drought tolerance, and reduced water loss in transgenic tomato plants [41].

Polyploid plants with a larger genome can produce more cuticular wax than diploid plants, improving tolerance to environmental stresses [5]. Liu et al. [5] reported that two wax-related genes were upregulated in triploid mulberry (*Morus alba*) under drought conditions. These findings indicated that triploid plants exhibit a lower water loss rate than their diploid progenitors [5]. In the present study, in comparison with those in diploid leaves, the relative expression of two genes involved in wax biosynthesis, *CER1* and *SHN1*, was significantly greater in autotetraploid leaves under stress conditions, suggesting that these plants can accumulate cuticular wax and tolerate multiple abiotic stresses. In fact, cuticular wax production and deposition are affected by an enlarged genome that can increase the tolerance of polyploid plants [5]. The expression levels of these genes were greater in both diploid and tetraploid genotypes under stress conditions than under control conditions. These results indicate that *CER1* and *SHN1*, which are involved in cuticular wax accumulation and drought tolerance, are activated by drought. Similarly, Zhou et al. [42] reported that the waxy gene *Glossy1-3* (*OsGL1-3*) in rice, similar to *AtCER1* in Arabidopsis, was activated by drought.

In recent years, the biosynthetic pathways of cuticular wax compounds have been studied from a molecular point of view, and the genes and regulators involved in the biosynthesis of these compounds have been isolated and identified. There are many reports that alkanes are the most important components of cuticular wax in plants. These compounds play an essential role in controlling the permeability of plant cuticles, and their reduction leads to decreased tolerance to drought stress [5, 19, 43]. According to the report of Jetter and Kunst (2008), alkanes account for 70% of the total wax in leaves [44]. The *CER1* gene together with *CER3* are the key components of the alkane synthesis complex [19]. Bourdenx et al. [19] reported that overexpression of *CER1* led to a greater amount of total wax, which was largely due to the increase in the amounts of odd-carbon-numbered alkanes, mainly C29 alkanes. Lemieux et al. [45] indicated that the epicuticular wax of *CER1* mutants had lower amounts of alkanes, secondary alcohols, ketones, and aldehydes than did that of wild-type plants. According to the findings of Cao et al. [46], the *brCER1* mutant gene in nonheading chinese cabbage produced a wax-less phenotype. Wang et al. [47] reported that the overexpression of *CsCER1* reduced cuticle permeability and increased tolerance to drought in cucumbers. Djemal et al. [35] showed that overexpression of *TdSHN1* increases the expression of ROS and osmotic-related genes, such as *ERD* and genes involved in wax biosynthesis and *CER1*, which contribute to the formation of alkanes, as the major component of cuticular wax. Li et al. [14] demonstrated lower water loss rates and increased drought tolerance of autotetraploid *Ziziphus* plants. The enhanced cuticular wax properties was due to increased quantity of the alkanes [14]. Similarly, Li et al. [30] indicated that among the different wax constituents, C29 and C31 alkanes were the greatest compounds after drought stress. Investigation of wax compounds in the leaves of wallflowers revealed that alkanes (50%) accounted for the majority of the total cuticular wax composition of wallflower leaves, approximately 76% of which were related to C29 and C30 alkanes. Furthermore, there was a close correlation between alkane and fatty acid biosynthesis and between *CER1* and *SHN1* gene expression. A greater expression of the *CER1* and *SHN1* genes under stress conditions affected the alkane amount and total wax content.

The cuticular wax content of autotetraploid leaves was significantly greater than that of diploid wallflowers, which might be associated with the greater density of wax crystals on tetraploid leaves. Recent studies have reported that changes in the density and/or dispensation of wax crystalloids lead to alterations in the permeability of the cuticle and in the water loss rate [48]. An increase in cuticular wax content induced by drought is one of the strategies used by polyploid plants to prevent water loss [5]. Polyploid plants seem to activate several signaling pathways, leading to the synthesis and accumulation of a large number of alkanes, increasing the total wax content, and alleviating drought stress [37]. Djemal et al. [35] showed that overexpression of *TdSHN1* in transgenic tobacco plants led to chlorophyll leaching, water loss, and consequently decreased permeability. In contrast, *AtSHN1* overexpression in *Arabidopsis* results in wax accumulation, increased chlorophyll leaching and water loss rates, and increased drought tolerance [49]. Under water deficit conditions, the water loss rate (21%) in the tetraploid wallflowers was lower than that in their diploid counterparts (35%) after 300 min. However, there was a negligible difference between the drought-stressed and well-watered diploid plants as well as control tetraploids in terms of the water loss rate. The results of this study showed that higher expression of the *CER1* and *SHN1* genes in tetraploid wallflowers under stress conditions affected the alkane amounts as well as the total wax content and reduced the water loss rate. The reduced water loss rate in tetraploid wallflowers was affected by their greater wax content, which led to reduced cuticle permeability. This finding was in line with previous studies reporting that cuticular wax can reduce nonstomatal water loss to maintain high water potential in leaves [34]. Sajeevan et al. [38] reported that the wax content and morphology of wax crystals and their arrangement, as well as the biochemical constituents of cuticular waxes, affect the water loss rate and permeability of cuticles.

SEM analysis was used to evaluate the micromorphology of leaf cuticular wax in *E. cheiri.* Compared with diploid leaves, tetraploids exhibit a wide range of morphologies, including platelets, tubular clusters, and polygonal rodlet structures, under both water deficit and control conditions that might mediate the reduction in water loss and ameliorate drought stress. In wallflowers, the diverse shapes, higher densities, different architectures of wax crystalloids and different biochemical compositions can explain the distinctive whitish fluffy appearance of tetraploid leaves compared with diploids. Moreover, the density of wax platelets in tetraploid wallflowers was significantly greater than that in diploids. Our results are in line with those of Li et al. [14] (*Ziziphus jujuba*) and Liu et al. [5] *(Morus alba)*, who reported denser crystals of cuticular wax in tetraploid and triploid plants, respectively, than in diploid leaves. Taken together, although the molecular mechanisms of genome doubling in wax biosynthesis are yet unknown, polyploid plants under stress conditions seem to establish multigene networks and new metabolic and regulatory pathways to enhance plant adaptation.

## Conclusion

This study revealed the superiority of using tetraploid-induced wallflowers to tolerate water deficit stress. Due to genomic duplication, many genes were upregulated in autotetraploid plants compared to diploids under drought stress, leading to greater tolerance. In addition, the biosynthesis of cuticular wax as an epidermal barrier against water loss was studied morphologically, molecularly, and biochemically. In this study, a total of 4 cuticular wax compounds were identified. The alkanes (C29, C30, and C31), alcohols (C28 and C30), and aldehydes (C32) were the most prevalent constituents. Moreover, tetraploid wallflowers had the highest wax content and the lowest rate of water loss. The molecular mechanisms underlying the effect of duplicated genomes on drought tolerance improvement are not yet fully understood, and many hormonal, regulatory, and metabolic signaling pathways, as well as epigenetic mechanisms, are involved in this process synergistically. The findings of this study demonstrate a better picture for drought tolerance in polyploid plants.

### Electronic supplementary material

Below is the link to the electronic supplementary material.

Supplementary Material 1

## Data Availability

The accession numbers used in this study are available in the National Center for Biotechnology Information repository via the link https://www.ncbi.nlm.nih.gov. The accession No. are AREB1: AB017160.1, NM_179446.5, NM_001333260.1; AREB3: XM_023782262.1, XM_010506292.2, NM_115544.3; ERD1: NM_001084956.2, XM_024149664.1, XM_019231546.1, XM_023779447.1; RD29A: XM_023779408.1, XM_010484279.2, NM_124610.3; CER1: XM_019242739.1, XM_023777524.1, NM_100101.4, XM_024153181.1; SHN1: XM_010498787.2, XM_006305563.2, NM_101405.4; ACTIN2: NM_001302975.1, XM_010508838.2 and XM_013829442.2.
