# Supplementary material for: Gene expression analysis of drought tolerance and cuticular wax biosynthesis in diploid and tetraploid induced wallflowers
Source: BMC Plant Biol. 2024 Apr 25;24:330. doi: 10.1186/s12870-024-05007-6 (PMC11044323; doi:10.1186/s12870-024-05007-6)
Supplement: Supplementary file 1 — Supplementary Material 1 [file 12870_2024_5007_MOESM1_ESM.docx]

**Table S1.** Sequences of primers used for real-time PCR analysis in *E. cheiri.*

| **Primer Name** | **Primer Sequence (5ʹ to 3ʹ)** | **Length (bp)** | **Annealing Temperature (°C)** |
| --- | --- | --- | --- |
| *AREB1*-F | AACAACATACCAGCAATCG | 19 | 60 |
| *AREB1*-R | GACCACCACCTCTTATCC | 18 |  |
| *AREB3*-F | TCAATGCCACAGCCTCAA | 18 | 63 |
| *AREB3*-R | ACAGTCTTCTCTACAACTTCTCC | 23 |  |
| *RD29A*-F | AGAAGAGAAGAAGGAAAC | 18 | 56 |
| *RD29A*-R | CCGAACCATCCTTTAATC | 18 |  |
| *ERD1*-F | CCTTCTTGGTTAAGACTGA | 19 | 57 |
| *ERD1*-R | CTCTCGTATTCTTGATTGTATA | 22 |  |
| *CER1*-F | CATCGCCTCTGCTCTCTG | 18 | 63 |
| *CER1*-R | CTTCTCCCACCAGCCATAC | 19 |  |
| *SHN1*-F | ATTACTCATCATCAAGTTCCTA | 22 | 58 |
| *SHN1*-R | CCAAATCCTCCGTTTCAA | 18 |  |
| *ACTIN2*-F | CTGGATTCTGGTGATGGT | 18 | 60 |
| *ACTIN2*-R | GCTTCTCCTTGATGTCTCT | 19 |  |

**
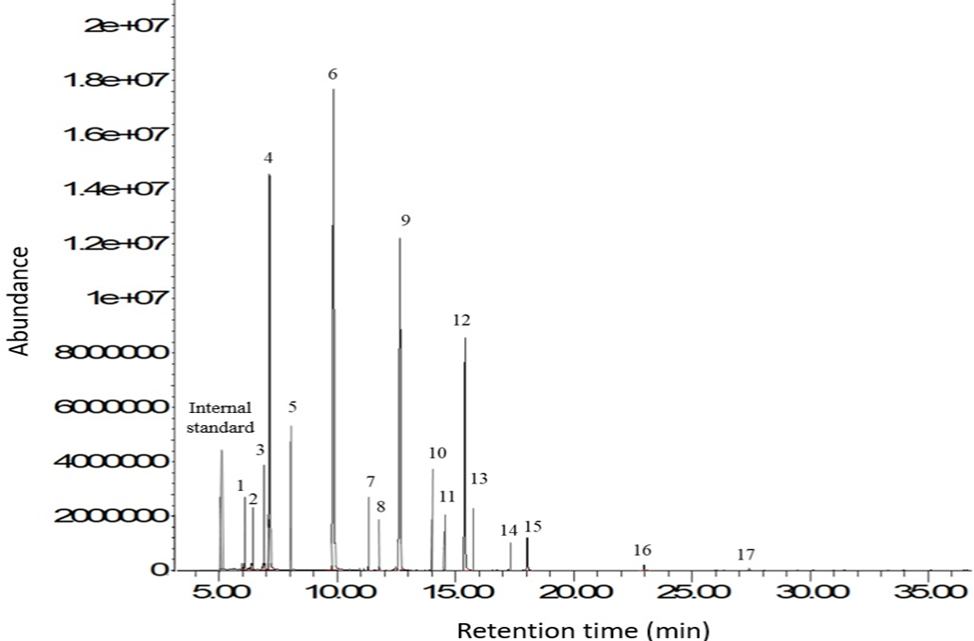
**

**Fig. S1** GC–MS chromatograms of the chemical compositions of cuticular wax components from wallflower leaves. The internal standard was hexadecane. 1) C27 alkane, 2) C28 alkane, 3) C24 alcohol, 4) C29 alkane, 5) C28 aldehyde, 6) C30 alkane, 7) C26 alcohol, 8) C30 aldehyde, 9) C28 alcohol, 10) C31 alkane, 11) C32 alkane, 12) C32 aldehyde, 13) C33 alkane, 14) C26 fatty acid, 15) C30 alcohol, 16) C28 fatty acid, 17) C30 fatty acid.
